# Results of Intrathecal Baclofen Treatment in Sixteen Spasticity Patients According to Four Different Measurement Scales: A Retrospective Analysis

**DOI:** 10.7759/cureus.26980

**Published:** 2022-07-18

**Authors:** Hasan Burak Gündüz

**Affiliations:** 1 Neurological Surgery, Bakirkoy Prof. Dr. Mazhar Osman Training and Research Hospital for Psychiatric Neurological Diseases, Istanbul, TUR

**Keywords:** spasticity, modified functional ambulation classification, visual analogue scale, modified ashworth scale, penn spasm frequency scale, intratechal baclofen

## Abstract

Introduction

Spasticity is a motor disorder characterized by a velocity-dependent increase in tonic stretch reflexes. It occurs as a result of overstimulation of the stretch reflex and is a component of the upper motor neuron syndrome. Intrathecal Baclofen (ITB) pump administration in patients with a diagnosis of spasticity may be a suitable option for reducing the complaints of the patients and increasing their quality of life. The aim of this study is to analyze clinically and statistically the diagnosis, treatment criteria, and post-treatment results of patients with spasticity who were treated in our clinic.

Materials and Method

Sixteen patients who were diagnosed with spasticity and placed on an intrathecal Baclofen pump between January 2015 and December 2020 were included in this study. An intrathecal Baclofen trial was first applied to patients who were candidates for the Baclofen pump. The spasticity levels of the patients who decided to have an intrathecal Baclofen pump were scored according to the modified Ashworth scale (MAS) and Penn spasm frequency scale (PSFS). In addition, the scaling of the patients' own conditions according to the visual analogue scale (VAS) and ambulation status according to the modified functional ambulation classification (MFAC) were recorded. All these evaluations were repeated in the preoperative, early postoperative, and follow-up periods.

Results

The sex distribution of the patients included in the study was equal to eight women and eight men. The age distribution was between 18 and 76. The average age was 40.62 (standard deviation ±17.79). The average preoperative modified Ashworth scale score was 3.73, and the average Penn spasm frequency scale score of the patients was 3.67. The average preoperative modified functional ambulation classification score was 1.87, and the average visual analogue scale score was 6.67. At the end of the second postoperative week, the average modified Ashworth scale score was 1.80 and the average Penn spasm frequency scale score was 1.67. The modified functional ambulation classification score was 2.60 and the visual analogue scale score was 4.58. The average follow-up period of the patients was 64 months. At the end of the follow-up periods, the average late-period modified Ashworth scale score was 1.87, and the Penn spasm frequency scale score was 1.67. The average modified functional ambulation classification score was 3.00, and the average visual analogue scale score was 4.50. Statistically, there was a significant difference between preoperative and postoperative results in both modified Ashworth scale and Penn spasm frequency scale scores (P<0.05). Modified functional ambulation classification preoperative and postoperative comparison results (P<0.05) and visual analogue scale results (P<0.05) were also statistically significant. No significant difference was found between the early postoperative period and the late postoperative period in all measurements (P=1.00).

Conclusion

Intrathecal Baclofen administration is one of the many treatment options for spasticity. In this way, it has been shown that greater Baclofen efficacy is achieved and its side effects are reduced. It should always be remembered that the process of this treatment is teamwork that requires the participation of more than one specialty branch. Physical therapists, neurologists, pediatricians, and neurosurgeons should be included in this teamwork.

## Introduction

The aim of this study is to investigate the effectiveness of intrathecal Baclofen (ITB) treatment by examining the preoperative and postoperative objective and subjective data in patients diagnosed with spasticity.

Spasticity is a motor disorder characterized by tight or stiff muscles that may interfere with voluntary muscle movements and is observed in many patients with spinal cord injury, multiple sclerosis, cerebral palsy, and acquired brain injury [1,2]. This clinical picture refers to a cluster of clinical signs that include increased tone, hyperactive reflexes, weakness, and poor coordination. These clinical manifestations are believed to result from the loss of inhibitory suprasegmental inputs that produce hyperactive segmental spinal reflex arcs [3-5].

The role of Baclofen in the treatment of spasticity

Baclofen is an agonist of the inhibitory neurotransmitter ɣ-aminobutyric acid (GABA) and is structurally 4-chloro-phenyl-GABA. Baclofen in drug form is a racemic mixture and acts on GABAa receptors throughout the central nervous system to inhibit the release of excitatory neurotransmitters such as glutamate and aspartate by inhibiting the required calcium uptake. According to Albright, the effect of Baclofen on spasticity is probably through the L-enantiomer, which acts on bicuculline-insensitive GABAb receptors located superficially in the II and III Rexed layers of the spinal cord [4,5]. Baclofen has no direct effect on peripheral muscles [6-9]. However, orally administered Baclofen has some supraspinal activities in the brain that may cause clinical side effects. The main side effects of oral Baclofen are sedation, extreme weakness, dizziness, mental confusion, and drowsiness [2]. The incidence of side effects has been reported to vary between 10% and 75% [2,10].

ITB treatment increases the inhibitory effects of GABAb receptors in the spinal cord and eliminates the side effects of oral antispasmodic agents [11,12]. Continuous intrathecal administration of Baclofen via an intracorporeal pump system was first used in 1984 by Penn and Kroin to treat adult patients with spasticity. The treatment of a spastic child with an ITB pump was first performed by Dralle et al. in 1985 [13-15].

## Materials and methods

Sixteen patients who were diagnosed with spasticity and received an ITB pump between January 2015 and December 2020 were included in this study. All patients were operated on in the Neurosurgery Clinic of Istanbul Bakırköy Prof. Dr. Mazhar Osman Research and Training Hospital for Neurology, Neurosurgery, and Psychiatry. The required ethics committee approval number is 2021-03-03, and the approval date is February 01, 2021.

Patient selection

All candidate patients were evaluated by a physical therapist, a neurologist, and a neurosurgeon. The patients were followed up by these three expert groups during and after the surgical intervention.

The accepted criteria for a patient to be a candidate for an ITB pump were: (1) presence of global or regional spasticity (±dystonia); (2) a modified Ashworth scale (MAS) or Penn spasm frequency scale (PSFS) score > 2; (3) spasticity affecting two or more limb regions, including both lower and/or one or both upper limbs, either unilaterally or bilaterally; and (4) history of spasticity longer than six months [16].

An ITB trial is first applied to patients who are candidates for an ITB pump. Preferably, 50 µg (diluted as 50 µg/ml) Baclofen is administered intrathecally as a bolus at the L3-L4 level. The result is monitored with a 24-hour follow-up. If suboptimal results are obtained, the dose can be increased to 75 µg and then to 100 µg.

Before the injection and 2, 4, 6, 12, and 24 hours after the trial dose of ITB, muscle tone assessments of the hip flexors, hip adductors, knee flexors, knee extensors, and ankle plantar flexors were performed by a physical therapist using MAS (Table 1) and PSFS (Table 2) [17,18]. Based on this review, the decision to perform ITB surgery is made upon observation of objective decreases of >1 in the MAS score or >1 in the PSFS score in at least two affected limb areas after a trial dose of ITB. It is concluded that the patient will benefit from the ITB application [16]. Patients for whom surgical intervention was not decided as a result of the ITB trial and patients under the age of 18 were excluded from the study. The reason for the exclusion of those under the age of 18 is the decision of the ethics committee.

**Table 1:** Modified Ashworth scale for grading spasticity

Grade	Description
0	No increase in muscle tone
1	Slight increase in muscle tone, manifested by a catch and release or by minimal resistance at the end of the range of motion (ROM) when the affected part(s) is moved in flexion or extension
+1	Slight increase in muscle tone, manifested by a catch, followed by minimal resistance throughout the remainder (less than half) of the ROM
2	More marked increase in muscle tone through most of the ROM, but affected part(s) easily moved
3	Considerable increase in muscle tone, passive movement difficult
4	Affected part(s) rigid in flexion or extension

**Table 2:** Penn spasm frequency scale

Spasm score	Frequency of spasms
0	No spasms
1	Mild spasms induced by stimulation
2	Infrequent full spasms occurring less than once per hour
3	Spasms occurring more than once per hour
4	Spasms occurring more than 10 times per hour

Surgical process

Under general anesthesia, the patient is positioned as lateral decubitus with the operative side up. The right side is preferred unless there is a contraindication. Fluoroscopy is brought to the surgical site under sterile conditions. The purpose of fluoroscopy is primarily to confirm the entry-level as well as to determine the level of the catheter delivered into the intrathecal distance. The preferred area between L1 and L5 is prepared for catheter entry. Generally, the L3-L4 level is preferred.

The intrathecal catheter is introduced into the subdural space up to the planned vertebral level. The level of the delivered spinal catheter is determined according to the targeted clinical goals. If the target is only the lower extremities, it is sufficient to send it up to the T8-T10 level. If the aim is to provide relaxation in the upper extremities, T3-T6 levels are preferred. The level at which the catheter is sent and whether there is any wrong position is checked by fluoroscopic control.

A surgical incision is made between the costal margin and the iliac crest as far as the pump can enter. Under standard conditions, the pump is placed in the subcutaneous fat tissue. The intrathecally inserted catheter is connected to the pump through a subcutaneous tunnel. The pump is attached to the abdominal muscle fascia. It is checked whether the system is working or not. Infusion is started at the planned dose. All surgical incisions are closed.

The pump is filled and the drug infusion is started. The initial infusion rate is 50 μg/day for all patients. The infusion rate is gradually increased until clinical improvement is seen. In this process, drug side effects are also monitored. During this period, oral administration is gradually reduced. An acute withdrawal syndrome should also be avoided at this time. Minimal physical therapy is also applied in the early postoperative period.

Clinical evaluation

The spasticity levels of the patients who underwent ITB pumps were graded according to MAS and PSFS. During this evaluation process, different muscle groups in the upper and lower extremities were examined. In addition, scaling of patients' own status according to the visual analog scale (VAS) and ambulation status according to modified functional ambulation classification (MFAC) were recorded (Table 3) [19,20].

**Table 3:** Modified functional ambulation classification

Categories	Stage	Definition
I	Lyer	Patient cannot ambulate and requires manual assistance to sit, or is unable to sit for 1 minute without back or hand support, with the bed or plinth height allowing hips, knees, and ankles positioned at 90° and both feet flat on the floor.
II	Sitter	Patient is able to sit for 1 minute without back or hand support and is unable to ambulate with the help of only one person.
III	Dependent walker	Patient requires manual contacts of no more than one person during ambulation on level surfaces to prevent falling. Manual contacts are continuous and necessary to support body weight as well as to maintain balance and/or assist coordination.
IV	Assisted walker	Patient requires manual contacts of no more than one person during ambulation on level surfaces to prevent falling. Manual contacts are continuous or intermittent light touch is required to assist balance and/or coordination.
V	Supervised walker	Patient can ambulate on level surfaces without manual contact of another person, but for safety reasons, he/she requires standby guarding or verbal cuing of no more than one person
VI	Indoor walker	Patient can transfer, turn and walk independently on level ground, but requires supervision orphysi cal assistance to negotiate any of the following: stairs, inclines, or uneven surfaces.
VII	Outdoor walker	Patient can ambulate independently on level and non-level surfaces, stairs, and inclines.

Statistical analysis

For statistical analysis, SPSS version 16.0 was used. All data were subjected to the Shapiro-Wilk test of normality. Since the skewness and kurtosis results of all the data were between −1, 5 and +1.5, it was concluded that the data distribution was normal. Preoperative, postoperative early and late MAS, PSFS, MFAC, and VAS scores were measured and compared. Preoperative, early postoperative, and late follow-up results were evaluated using the repeated measures ANOVA test.

## Results

Patient information

As a result of the trial, 16 of 19 patients who were referred to our clinic for ITB administration were found to be suitable for pump insertion. The sex distribution of the patients with an ITB pump was equal to eight females and eight males. The age distribution was between 18 and 76. The average age was 40.62 (standard deviation [SD] ±17.79). In the etiology of spasticity, there were four spinal traumas, three cerebral palsy, two multiple sclerosis, one spinal artery occlusion, one pott abscess, one central nervous system infection, one hereditary spastic paraplegia, one spondylotic myelopathy, one cerebrovascular accident, and one cerebral hypoxia due to cardiac arrest. The time between the onset of the disease and the date of ITBP insertion ranged from 1 year to 32 years. All patients were referred by physical therapy and neurology clinics. On the neurological examination of the patients, six patients were quadriparetic, four patients were quadriplegic, four patients were lower extremity paraplegic, one patient was upper extremity monoparetic, and one patient was lower extremity monoparetic. Although the level of the ITB catheter varied partially, it was adjusted according to the spasticity localization of the patients (Tables 4-5).

**Table 4:** Sex, age, duration of primary disease, and follow-up times

Patients	Sex	Age	The duration of the primary disease (year)	Follow up (month)
1	F	23	23	60
2	M	30	1	78
3	M	67	4	71
4	M	76	1	System was removed
5	F	22	1	66
6	M	37	26	65
7	F	18	18	61
8	F	27	3	57
9	F	61	21	53
10	F	40	30	126
11	F	39	17	222
12	M	34	34	30
13	M	71	13	30
14	M	38	19	16
15	M	25	3	15
16	F	42	15	10

**Table 5:** Etiology, neurological findings and intratechal Baclofen pump catheter tip levels of 16 patients

Patient	Etiology	Neurological findings	Level of the catheter tip
1	Cerebral palsy	Quadriplegic	T4
2	Cervical trauma (gunshot injury)	Quadriparetic	T4
3	Cervical spondylosis (progressive myelopathy)	Quadriparetic	T5
4	Cervical trauma	Quadriparetic	System was removed
5	Spinal artery occlusion	Quadriparetic	T6
6	Central nervous system infection	Quadriparetic	T6
7	Cerebral palsy	Quadriplegic	T6
8	Thoracolumbar spinal trauma	Lower extremity paraplegic	T5
9	Multiple sclerosis	Lower extremity monoparetic	T9
10	Hereditary spastic paraplegia	Lower extremity paraplegic (four extremity spastic)	T5
11	Spinal infection	Lower extremity paraplegic	T6
12	Cerebral palsy	Lower extremity paraplegic	T8
13	Cerebrovascular accident	Quadriparetic	T6
14	Cervical trauma	Quadriplegic	T6
15	Hypoxic ischemic encephalopathy	Quadriplegic	T6
16	Multiple sclerosis	Upper extremity monoparetic (four extremity spastic)	T6

Scoring of results

The average preoperative MAS score of the patients was 3.73, and the PSFS score was 3.67. The average preoperative MFAC score was 1.87, and the VAS score was 6.67. Patients were examined at 6, 12, 24, and 48 hours after surgery. Baclofen doses were adjusted. The final infusion doses decided in the postoperative period were a minimum of 50 and a maximum of 400 µg/day. The average dose administered to all patients was 122.67 µg/day.

At the end of the second postoperative week, the average MAS score was 1.80 and the average PSFS score was 1.67. The FMAC score was 2.60 and the VAS score was 4.58. These data were considered early postoperative results. In one patient, the pump system was completely removed in the third month as an infection developed in the operation area in the postoperative period. Therefore, the inclusion of data for this patient in the average was abandoned.

**Figure 1:**
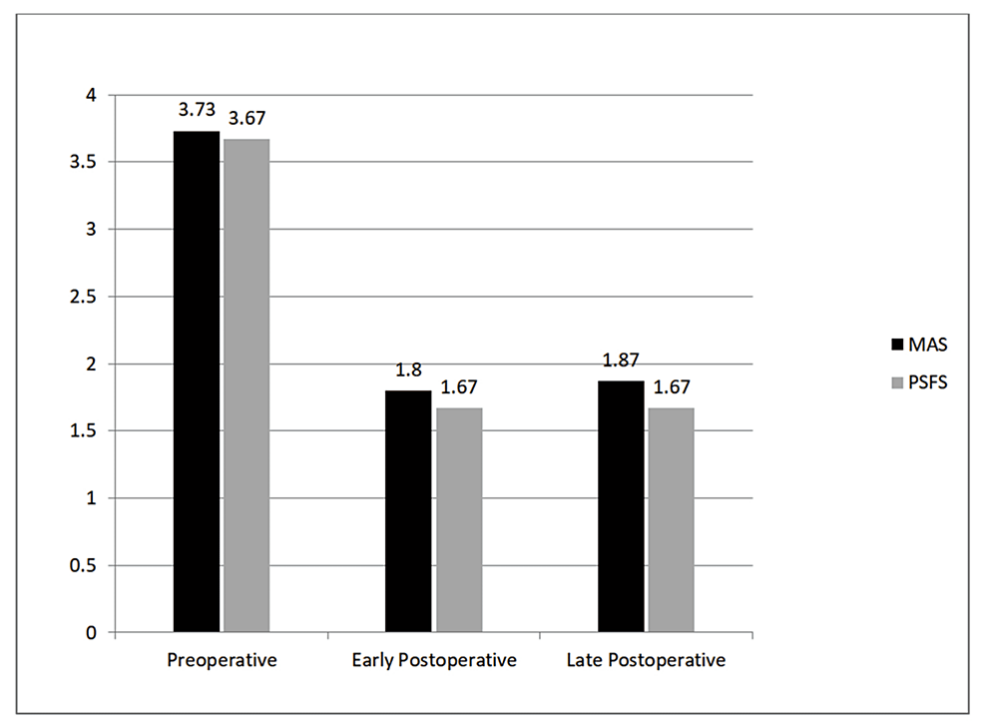
Preoperative, postoperative, and late follow-up modified Ashworth scale and Penn spasm frequency scale average values MAS: modified Ashworth scale, PSFS: Penn spasm frequency scale

**Figure 2:**
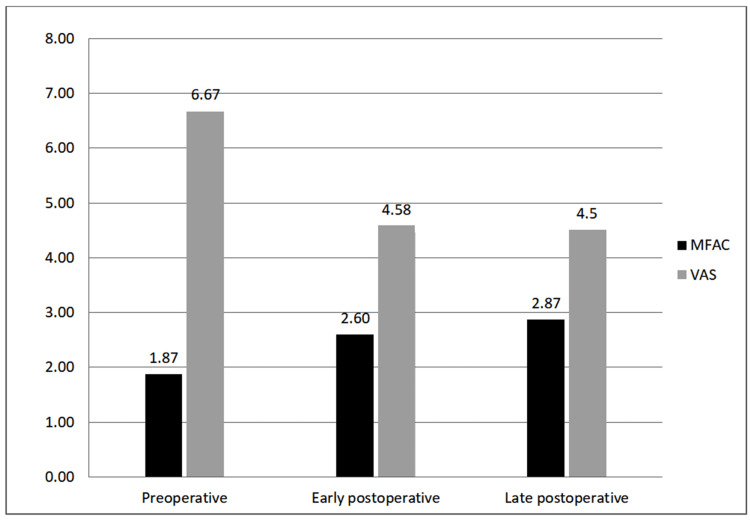
Preoperative, early postoperative, and follow-up modified functional ambulation classification and visual analog scale scores MFAC: modified functional ambulation classification, VAS: visual analog scale

The average follow-up period of the patients was 64 months (SD±51.11). Follow-up periods ranged from 10 months to 222 months. The pumps of a patient with a follow-up of 222 months were changed twice, and the pumps of two patients with a follow-up of 126 months and 78 months were changed once. The ITB pump system of a patient whose pump had been non-functional for 10 years was completely replaced. The previous period was not included in the follow-up period. One patient did not approve of pump replacement at the end of the 60th month. Doses of Baclofen were increased in three patients whose pumps were changed, considering that there was a decrease in efficacy. The Baclofen dose was increased from 115 µg/day to 125 µg/day in one patient and from 75 µg/day to 100 µg/day in two patients. At the end of the follow-up periods, the average late-period MAS score was 1.87, and the PSFS score was 1.67 (Table 6 and Figure 1). The average MFAC score was 3.00, and the average VAS score was 4.50 (Table 7 and Figure 2).

**Table 6:** Follow-up times of the patients, Baclofen doses, preoperative, early postoperative and follow-up modified Ashworth scale and Penn spasm frequency scale scores. MAS: modified Ashworth scale; PSFS: Penn spasm frequency scale

Patient	Follow up (month)	Baclofen dose (µg/day)	Preoperative MAS	Postoperative early period MAS	Late period MAS	Preoperative PSFS	Postoperative early period PSFS	Late period PSFS
1	60	120	4	2	2	4	2	2
2	78	115	4	2	2	4	3	2
3	71	50	4	2	2	3	1	1
4	The pump system was completely removed due to surgical site infection that did not respond to treatment.							
5	66	75	4	3	3	4	0	1
6	65	50	3	1	1	4	0	0
7	61	150	3	3	3	4	4	3
8	57	150	4	2	2	4	4	4
9	53	150	4	1	1	4	1	1
10	126	75	4	2	2	3	2	2
11	222	75	4	2	2	4	1	1
12	30	75	3	2	2	4	2	2
13	30	150	3	1	1	3	1	2
14	16	400	4	1	1	4	1	1
15	15	80	4	1	2	3	1	1
16	10	125	4	2	2	3	2	2
Average	64	122.67	3.73	1.80	1.87	3.67	1.67	1.67

**Table 7:** Preoperative, early postoperative, and follow-up modified functional ambulation classification and visual analog scale scores MFAC: modified functional ambulation classification, VAS: visual analog scale

Patient	Preoperative MFAC	Postoperative early period MFAC	Late period MFAC	Preoperative VAS	Postoperative early period VAS	Late period VAS
1	1	2	2	7	4	5
2	2	2	3	7	5	4
3	3	4	5	6	5	4
4	The pump system was completely removed due to surgical site infection that did not respond to treatment.					
5	3	4	5	6	4	4
6	2	3	3	7	4	4
7	1	1	2	Could not be measured	Could not be measured	Could not be measured
8	1	1	1	8	7	7
9	2	4	4	6	4	4
10	1	2	2	Could not be measured	Could not be measured	Could not be measured
11	1	2	2	7	4	4
12	3	4	4	7	5	5
13	3	4	5	5	3	3
14	1	1	2	8	5	5
15	1	1	1	Could not be measured	Could not be measured	Could not be measured
16	3	4	4	6	5	5
Average	1.87	2.60	3.00	6.67	4.58	4.50

Drug side effects and the other complications

In this study, drug-related side effects occurred as somnolence in one patient, constipation in one patient, and headache in one patient. Somnolence was corrected with early dose adjustment. Constipation and headache, on the other hand, responded to medical treatment in the course of time.

Complications related to the pump were infection at the surgical site in one patient, catheter occlusion in one patient, and bacterial meningitis in one patient. In the patient who had an infection in the operation area, anti-infection treatment methods were applied, but no response was obtained. Considering the old age of the patient, the pump and catheter were completely removed in the 3rd month postoperatively. In the patient who developed bacterial meningitis, the pump and catheter were removed, the meningitis was treated, and the ITB pump was reinserted. Since the patient with catheter occlusion did not come to the controls, the period of out-of-control was excluded from the follow-up period. When he applied again, the ITB pump system was completely replaced (Table 8).

**Table 8:** Postoperative complications and dose-related side effects

Complications		%
Dose-related side effects		
Somnolence	1	
Constipation	1	
Headache	1	
Total	3	18.75
Mechanical implant dysfunction		
Infection at the surgical site	1	
Bacterial meningitis	1	
Catheter occlusion	1	
Total	3	18.75

## Discussion

Selected evaluation scales

Different scales are used in the rating of spasticity. One of the most frequently used of these is MAS. The PSFS, modified Tardieu scale, and Rekand disability and spasticity scores are also used in the evaluation of spasticity [21]. Patients with ITB pumps were evaluated using four criteria: (1) MAS: It is a scale created to measure the severity of spasms. (2) PSFS: It provides the measurement of the frequency of spasms. These two scales provide objective measures of spasticity. They were included in this study in order to demonstrate the objective effectiveness of the treatment. (3) MFAC: based on the measurement of patients' ambulation capacity. MFAC was included in the evaluations as a criterion for participating in active daily life. Reduction in spasticity alone does not improve the patient's quality of life. Certain levels of spasticity may be beneficial in patients' ambulation [22]. Therefore, this classification was included in our evaluation criteria in order to measure the patient's ambulation function rather than only measure the spasticity level. (4) VAS: This classification is subjective, and the patient's statement is essential. The reason why VAS was chosen as an evaluation criterion is its subjective nature. Thus, the opinions of the patients could be added to the results.

Statistical assessment

Postoperative outcomes were classified as early and late periods. These results were first subjected to the Shakiro-Wilk test of normality. Then, preoperative, postoperative early, and late follow-up results were evaluated using the repeated measures ANOVA test. Accordingly, there was a significant difference between preoperative and postoperative results in both MAS and PSFS scores (P<0.05). MFAC preoperative and postoperative comparison results (P<0.05) and VAS results (P<0.05) were also statistically significant. No significant difference was found between the early postoperative period and the late postoperative period in all measurements (P=1.00).

Results and brief literature comparison

Preoperative, early postoperative, and late postoperative outcomes were compared in the light of MAS, PSFS MFAC, and VAS scores. When the proportional changes in the scores were calculated, it was observed that there was an improvement of 51.74%, 54.49%, 39.04%, and 31.33% in the MAS, PSFS, MFAC, and VAS scores in the early postoperative period, respectively. Compared to the postoperative early period, there was an improvement of 15.38% and 1.7%, respectively, in the MFAC and VAS score averages in the postoperative late period results. A partial worsening of the average MAS (3.89%) was observed between the early postoperative period and the late period. The average PSFS score did not change.

It is noteworthy that the positive changes in MFAC and VAS after ITB administration lag behind MAS and PSFS. At the same time, the evaluation of MFAC, which indicates the ambulation function, and VAS, which indicates the patient's opinion, together with the two basic criteria of spasticity examination, MAS and PSFS, reveals the general condition of the patients more clearly. In addition, although there was a partial regression in the MAS score between the early postoperative period and the late postoperative period, and the PSFS score did not change, the minimal positive change in MFAC and VAS scores strengthens this view. It is important to emphasize the contribution of physical therapy exercises that can be done more effectively after ITB in this recovery.

When the postoperative changes of the patients are compared with the literature data, it is seen that the results are compatible with the existing literature. According to the table obtained by the Medical Advisory Secretariat from Sampson et al., the average Ashworth score, which was 3.9 before ITB, decreased to 1.6 after ITB [2,23]. In the study of Heetla et al., the mean Ashworth score at baseline (before pump implantation) was 3.7. Ashworth scores decreased to a mean of 2.4 in all patients immediately after pump implantation. After six months and throughout the entire study, it remained below baseline [24].

Dose increase

In three of the patients in this series, the Baclofen dose was increased. The Baclofen dose was increased from 115 µg/day to 125 µg/day in one patient and from 75 µg/day to 100 µg/day in two patients. These increases were made during pump replacement. While the average dose of Baclofen was 122.67 in the early postoperative period, it was 126.67 in the late period. However, when this result was evaluated with the repeated measures ANOVA test, the increase in Baclofen dose was not found to be statistically significant (P=0.178). In the article by Skoog and Hedman, it is reported that the dosages of both spinal cord injury and multiple sclerosis patients are increased at varying rates. Similarly, in the article by Plassat et al., it is mentioned that there may be a need to increase Baclofen doses. It is stated that this increase may be due to tolerance to Baclofen [25,26]. In the study by Sampson et al., it was reported that Baclofen dosage should be increased during follow-up to reduce spasticity. The average starting dose was around 150 µg/day and, after 16 months, this increased by approximately 250% [23]. In the article by Heetla et al., the mean dose increased significantly during the first 18 months and stabilized afterward at 350 mg. The increases in the Baclofen dose in the three different patient groups (MS, SCI, and other causes) did not show any significant difference (P = 0.558) [24]. In the multicenter study of Taira et al., it was reported that drug tolerance was seen in only one patient [27].

In this study, dose change rates appear to be significantly lower than those reported in other articles. This result can be explained in two ways: first of all, the follow-up periods in this retrospective study are very variable (10-222 months). This variability may have prevented periodic patient follow-ups and monitoring of Baclofen doses. Second, since ITB pump application is a new field in our clinic, it is possible that our study group administering the treatment was conservative in dose increases to prevent complications. However, despite this self-criticism, it should be noted that, apart from an insignificant increase in the MAS average score, there was no worsening in the scores.

Drug side effects and complications

Complications arising from ITB are collected into two main groups: (1) dose-related complications and (2) mechanical implant dysfunctions [28].

In this study, drug-related side effects occurred as somnolence in one patient, constipation in one patient, and headache in one patient (18.75%). Somnolence was corrected with an early dose adjustment. Constipation and headache, on the other hand, responded to medical treatment in the course of time. Complications related to the pump were infection at the surgical site in one patient, catheter occlusion in one patient, and bacterial meningitis in one patient (18.75%). Surgical intervention was performed again in three patients (one in the late period) (18.75%).

In their multicenter study, Taira et al. listed the complications encountered due to the pump and catheter system as CSF leakage, infection, bending, bridging, occlusion, displacement, migration, pump rotation, alarm abnormality, memory error, delayed healing, malfunction, abnormal infusion rate, and surgical trouble [27]. Dysphagia, urinary incontinence, auditory hallucination, micturition difficulties, speech problems, drowsiness, somnolence, dizziness, blurred vision, nausea, vomiting, orthostatic hypotension, nystagmus, confusion, memory impairment, and dysmetria have been reported as drug-related side effects in various articles. Constipation and headache were rare side effects in the literatüre [26,28,29]. In the study of Heetla et al., the rate of drug-related side effects was calculated as 13.6% per year [24]. The overall percentage of catheter (8.5%), pump (1.8%), and infection (3%) problems reported in the multicenter studies of Taira et al. was 13.3%. Mild overdose symptoms were observed in 2.3% [27].

Catheter tip levels

During ITP pump application, the upper level of all catheters was limited in the thoracic region. The catheter tip level was tried to be adjusted according to the spasticity level of the patients. In patients with spasticity in the upper extremity, the upper limit of the catheter was removed up to thoracic 4 at most. The lowest level was thoracic 9. Generally determined level limits can be defined as the midthoracic and lower thoracic regions. Even in cases with the highest level of spasticity, catheter delivery to the cervical region was not preferred. The rationale for this is explained in the study by Grab et al. According to the study by Grab et al., placement of the catheter tip in the midthoracic region (T6-T7) results in a greater reduction in upper-extremity spasticity without loss of effect on the lower extremities despite lower Baclofen dosages when compared with lower thoracic (T12) catheter tip placement. A more rostral catheter position involves loss of trunk tone, causing greater difficulty in an upright posture. Also, Baclofen is more likely to cause supratentorial or brainstem side effects. At the current level, the catheter increases the risk of spinal cord injury [30].

Limitations of the study

The biggest limitation of this study is that pediatric patients were not included in the evaluation. This is because there is no pediatric clinic in our hospital. Therefore, the ethics committee excluded pediatric patients. In addition, if the number of our patients were greater, it would be possible to compare the patients according to their etiological origins. Finally, the highly variable follow-up period limited our observation of dose fluctuations during follow-up.

## Conclusions

Intrathecal Baclofen administration is one of the many treatment options for spasticity. In this way, it has been shown that greater Baclofen efficacy is achieved and its side effects are reduced. However, in this treatment process, the patients who will be administered intrathecal Baclofen should be chosen correctly. In addition, both drug effects and the surgical process should be closely monitored. Therefore, it should always be remembered that the process of this treatment is teamwork that requires the participation of more than one specialty branch. Physical therapists, neurologists, pediatricians, and neurosurgeons should be included in this teamwork.
